# On the Recognition of Natural Substrate CTP and Endogenous Inhibitor ddhCTP of SARS-CoV-2 RNA-Dependent RNA Polymerase: A Molecular Dynamics Study

**DOI:** 10.1021/acs.jcim.2c01002

**Published:** 2022-10-11

**Authors:** Angela Parise, Giada Ciardullo, Mario Prejanò, Aurélien de la Lande, Tiziana Marino

**Affiliations:** † Dipartimento di Chimica e Tecnologie Chimiche, Università Della Calabria, Via Pietro Bucci, 87036 Arcavacata di Rende, CS, Italy; ‡ Université Paris-Saclay, CNRS, Institut de Chimie Physique UMR8000, Orsay 91405, France

## Abstract

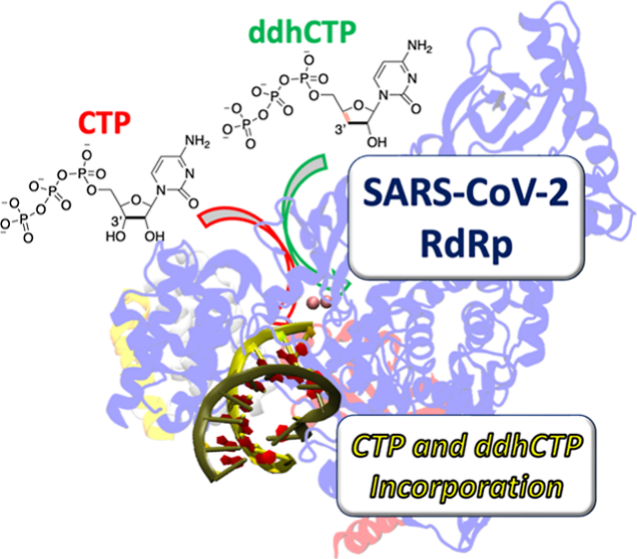

The novel coronavirus SARS-CoV-2 is the causative agent of the COVID-19 outbreak that is affecting the entire planet. As the pandemic is still spreading worldwide, with multiple mutations of the virus, it is of interest and of help to employ computational methods for identifying potential inhibitors of the enzymes responsible for viral replication. Attractive antiviral nucleotide analogue RNA-dependent RNA polymerase (RdRp) chain terminator inhibitors are investigated with this purpose. This study, based on molecular dynamics (MD) simulations, addresses the important aspects of the incorporation of an endogenously synthesized nucleoside triphosphate, ddhCTP, in comparison with the natural nucleobase cytidine triphosphate (CTP) in RdRp. The ddhCTP species is the product of the viperin antiviral protein as part of the innate immune response. The absence of the ribose 3′-OH in ddhCTP could have important implications in its inhibitory mechanism of RdRp. We built an in silico model of the RNA strand embedded in RdRp using experimental methods, starting from the cryo-electron microscopy structure and exploiting the information obtained by spectrometry on the RNA sequence. We determined that the model was stable during the MD simulation time. The obtained results provide deeper insights into the incorporation of nucleoside triphosphates, whose molecular mechanism by the RdRp active site still remains elusive.

## 1 Introduction

The world is currently in a state of pandemic emergency due to the spread of a disease called COVID-19 caused by a new viral etiologic agent, Severe Acute Respiratory Syndrome Coronavirus 2 (SARS CoV-2).^1^ Today, SARS-CoV-2 has become probably the fastest spreading virus in the human history. In fact, despite the vaccination, the continuous specific mutations occurring hide it from the immune system so that the use of antiviral therapy is mandatory to prevent future outbreaks of rapidly spreading coronaviruses.

In the fight against coronavirus, a great deal of computational studies on the molecular targets of SARS CoV-2 have been reported, and in particular, major efforts have been devoted to provide atomistic details of the spike (S) protein and proteases, which have received much attention from the computational chemistry community. An interested reader can find a fulfilling overview of excellent computational investigations in the recent review of Gao et al.^2^ However, very recently, interesting studies have appeared in the literature on RNA-dependent RNA polymerase (RdRp),^3,4^ which is the object of the present investigation.

In detail, SARS-CoV-2 is a positive RNA virus, which for its proliferation requires RdRp, a key enzyme that regulates the replication and transcription of the viral genome. For this reason, RdRp has become the validated target for the development of drugs against the COVID-19 disease, which can be hindered by the capacity of the human coronavirus to proofread^5^ and to remove mismatched nucleotides during genome replication and transcription. In fact, the incorporation of nucleoside analogue molecules into viral RNA can lead to increased mutation rates and to the failure of the virus through “lethal mutagenesis”.^6^ The RdRp complex, one of the largest proteins in the viral genome (932 residues), has multiple non-structural protein (nsp) units and, in particular, is organized in the nsp12 core catalytic unit, in the nsp7–nsp8 heterodimer, and in the nsp8 subunit, with a stoichiometry ratio of 1:1:2.^7^ These three non-structural proteins along with the nsp13 helicase are the “core” of the replication transcription complex. The category of antiviral drugs targeting the RdRp includes remdesivir,^8,9^ a nucleotide analogue adenosine mimic that showed efficacy against the Ebola infection and has been successfully repurposed for the treatment of SARS-CoV-2 infection such that it is the only drug approved against SARS-CoV-2 by the US Food and Drug Administration, on August 10, 2020.^10^ Several studies have proposed remdesivir to induce delayed chain termination^11−15^ or to cause the pausing and backtracking of polymerase, paving the way to the use of other nucleotide analogues.^16^

**Scheme 1:**
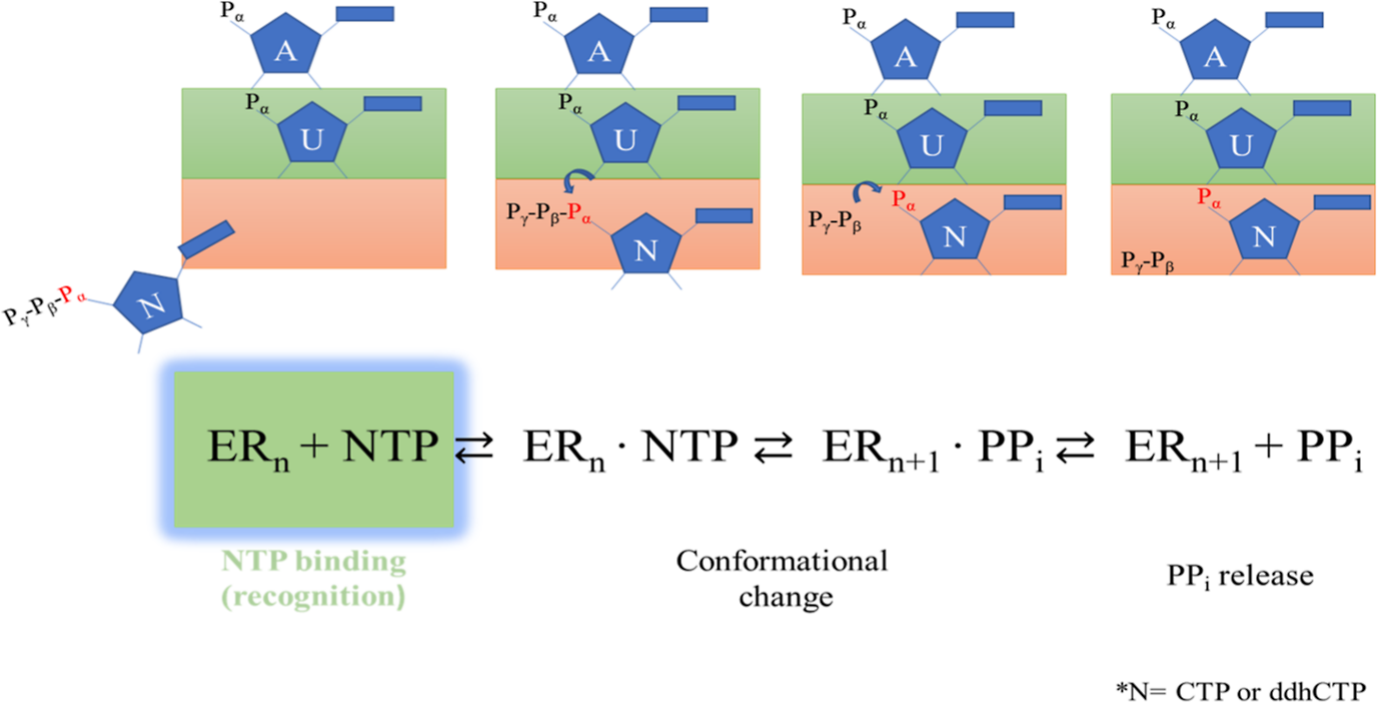
Canonical Schematic Reaction of RdRp

**Scheme 2:**
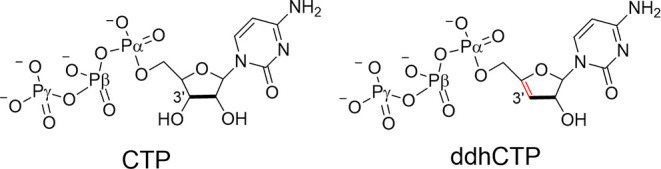
2D Representation of CTP and ddhCTP

3′-Deoxy-3′,4′-didehydro-cytidine triphosphate (ddhCTP), an analogue of CTP, is a novel antiviral nucleotide-like compound produced by enzyme viperin as part of the innate immune response,^17,18^ and its easy synthesis of ddhCTP has recently been described.^19^ Its chain terminator effect for RNA-dependent polymerases of multiple members of the Flavivirus genus has been further reported,^20^ and it has been recently demonstrated that the SARS-CoV-2 polymerase can incorporate this cytosine analogue well.^16^ Drawing inspiration from the evidence on ddhCTP^16,21^ and the computational work on remdesivir,^11,22^ a comparative in silico molecular modeling study (ddhCTP vs CTP) has been carried out on RdRp of SARS-CoV-2, to gain more insights into the incorporation of nucleoside triphosphates (see Scheme 1), which is one of the widely supported modes of interfering during viral replication pursuant to the high error rate (or low viral fidelity) of RNA virus replication.^5,23^ The comparison between these two species is interesting because ddhCTP competes with CTP,^24^ as confirmed from an intracellular concentration of ∼100 μM and lower than that of purine analogues,^25^ despite the difference of the −OH group loss in the 3′ position (see Scheme 2).

However, the absence of 3′-OH precludes further nucleotide incorporation from the RdRp enzyme, which is consequently stalled when the RNA chain moves through the polymerase and ddhCTP is processed. Another advantage of ddhCTP, with respect to other nucleoside analogue inhibitors, is that it does not require any particular metabolic transformations, differing from the case of other prodrugs (remdesivir and its derivatives).^16^

The choice of the in silico investigation of the endogenous product ddhCTP as an antiviral drug represents a good strategy to develop other compounds in more reduced times than those needed to obtain new therapeutic molecules from the scratch. Furthermore, ddhCTP represents an example of how the location of the modification in the ribose differently from that in other antiviral nucleotide analogues can affect the catalytic pathway used for incorporation.^16^

In the present study, classical molecular dynamics (MD) simulations are used to investigate the early steps of the RdRp working mechanism (recognition, see Scheme 1), including the contribution of possible conformational rearrangements in the substrate selection and binding.

Molecular docking has been used to test the affinity between the polymerase active site and the examined ligands. MD simulations were performed on the RdRp:RNA binary complex, including nsp7, nsp8, nsp12, and a partial double stranded RNA represented by a primer nucleotide strand of six units and a template strand of eight nucleotides, and the ternary complexes formed by RdRp:RNA with ddhCTP and CTP.

The comparative analysis of the dynamic behavior of RdRp in the presence of both the endogenously produced nucleotide analogue and chain terminator, ddhCTP, and the natural nucleotide CTP provides deeper insights useful for better discrimination of the factors underlying their incorporation in RdRp. In addition, the identification of amino acid residues important for the nucleotide recognition, and/or discrimination, represents one of the main focuses of the present investigation, coupled to the description at the atomistic level outer coordination shell of the two cations of the active site, which can be crucial for the viral replication.

## 2 Computational Methods

### 2.1 In Silico Built Model of RdRp:RNA

The investigation started from a cryo-EM^26^ crystal complex of RNA polymerase, with a resolution of 2.50 Å (PDB code 7AAP) and with a partial bound template primer RNA and RDV-TP. The crystallographic structure concerns the subunits nsp7 and nsp8 and the catalytic nsp12 bound to a template primer RNA duplex, in complex with favipiravir ribonucleoside triphosphate (favipiravir–RTP).^26^ Due to the different pyrimidine-like nature of ddhCTP with respect to that present in the above-mentioned crystallographic structure (favipiravir, a purine-like inhibitor),^26^ to build the proper biologically significant RNA fragment, a careful modeling process was required, coupling the crystallographic structural data and nucleotide sequence information obtained by mass spectrometry.^27^

Our study additionally includes the RNA template primer similarly to the work by Arba et al. on remdesivir,^22^ which is crucial for the nucleotide triphosphate binding, differently to other computational investigations on the remdesivir–SARS-CoV-2 RdRp interaction.^4,28−30^

Among the available crystallographic structures, our choice fell on the one that presented a complete structural composition (including the Zn cations, nsp7 and nsp8 domains) and the two magnesium ions essential for the RdRp activity, as very recently adopted.^4^ In addition, it is not always trivial to find out experimentally the number of Mg ions present in the active site as well as the identity and the number of their coordination ligands.^31^

**Figure 1:**
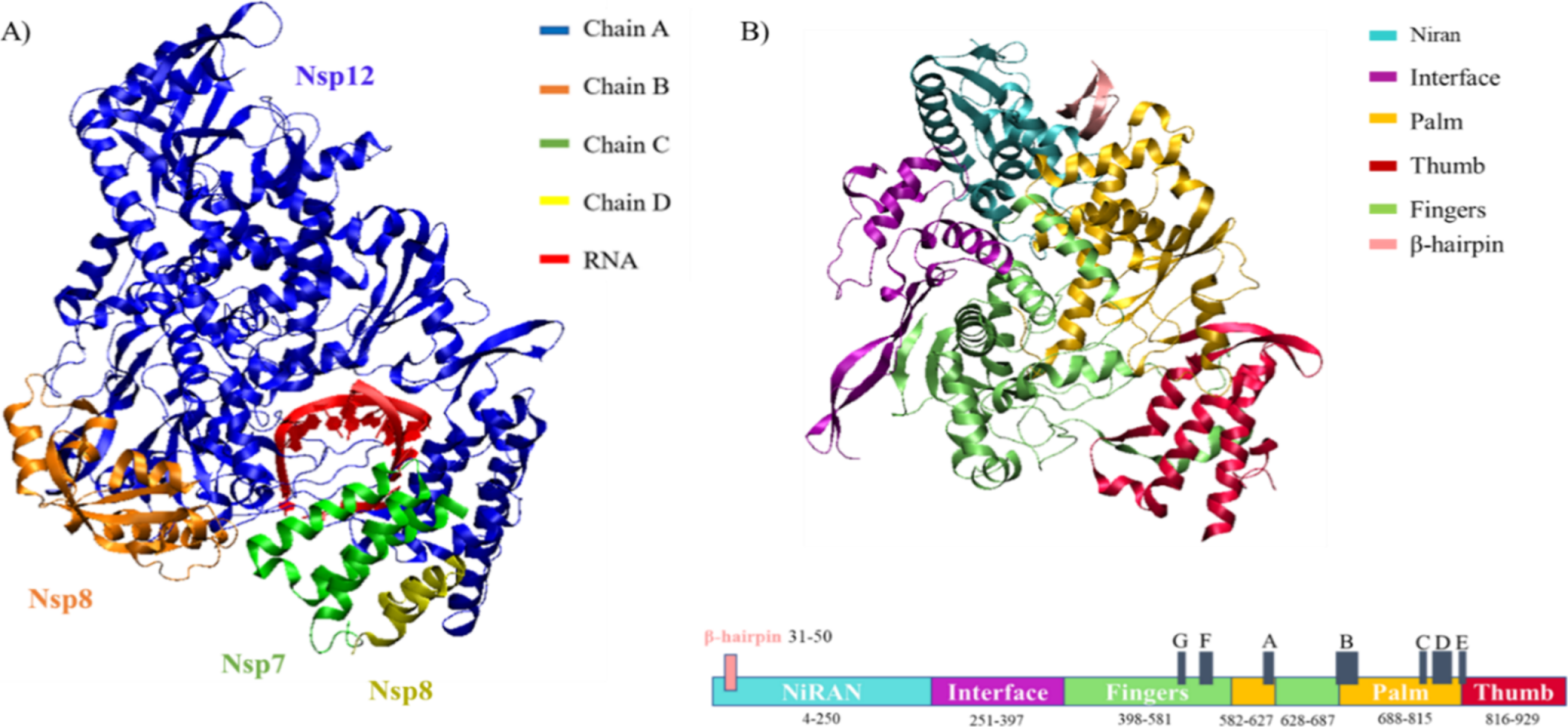
(A) Structures of the SARS-CoV-2 nsp7(2-68)-nsp8(78-191, 84-111)–nsp12(4-929) complex. (B) Structure of the nsp12 domain organized by color for subdomains and in gray for A–G motifs (motif A: s 612–626; motif B: residues 678–710; motif C: residues 753–767; motif D: residues 771–796; motif E: residues 810–820; motif F: residues 544–560; and motif G: residues 499–511).

The nsp12 structure contains a right-hand RdRp domain (residues Val398 to Thr929) and a specific N-terminal extension domain (residues from Asp60 to Ala250), which adopts a nidovirus RdRp-associated nucleotidyltransferase (NiRAN) architecture (see Figure 1).^32^

The polymerase and NiRAN domains are connected by an interface domain (residues Leu251 to Ser397). A further N-terminal β hairpin (residues Val31 to Lys50) inserts into the cleavage blocked by the NiRAN domain and the palm subdomain in the RdRp domain.

The polymerase domain adopts the conserved structure of the viral polymerase family^33^ and consists of three subdomains of a “cupped right hand”: a finger subdomain (residues Val398 to Ala581 and Asn628 to Thr687), a palm subdomain (residues Thr582 to Pro627 and Ala688 to Gln815), and a thumb subdomain (residues His816 to Thr929).

This right-hand architecture in turn evidences seven conserved structural motifs: motifs A–E located in the palm subdomain and motifs F and G in the finger subdomain.^34^ Most of these motifs are shared with other polymerases, indicating the importance of these structural elements in their enzymatic function.^35^

The active site, contained in the palm domain, is formed by residues Ser759, Asp760, and Asp761 of motif C.^36^

The carboxylate groups of these aspartates anchor a pair of divalent metal ions (Mg^2+^), which play the major role in catalysis,^33^ while two other metal ions (Zn^2+^) play a structural role to stabilize the enzyme. One of the zinc ions is coordinated to amino acid residues His295, Cys301, Cys306, and Cys310 in the N-terminal domain, while the second is bonded to Cys487, His642, Cys645, and Cys646 residues in the finger domain.^37^ As mentioned above, the selected RdRp crystal structure incorporates the favipiravir inhibitor, which is a purine nucleic acid analogue derived from pyrazine carboxamide (6-fluoro3-hydroxy-2-pyrazinecarboxamide).^38^

The design of the RNA double strand length and the type of bases for the cytosine analogue was then required. The selection of the RNA sequence has been based on matrix-assisted laser desorption ionization time-of-flight experiments on CTP-like RdRp inhibitors.^27^ The spectrometry study showed that the required base sequences for ddhCTP and CTP are UAAAAU (5′ ≥ 3′) and AUUUUAGU (3′ ≥ 5′), for the primer and template, respectively.

**Figure 2:**
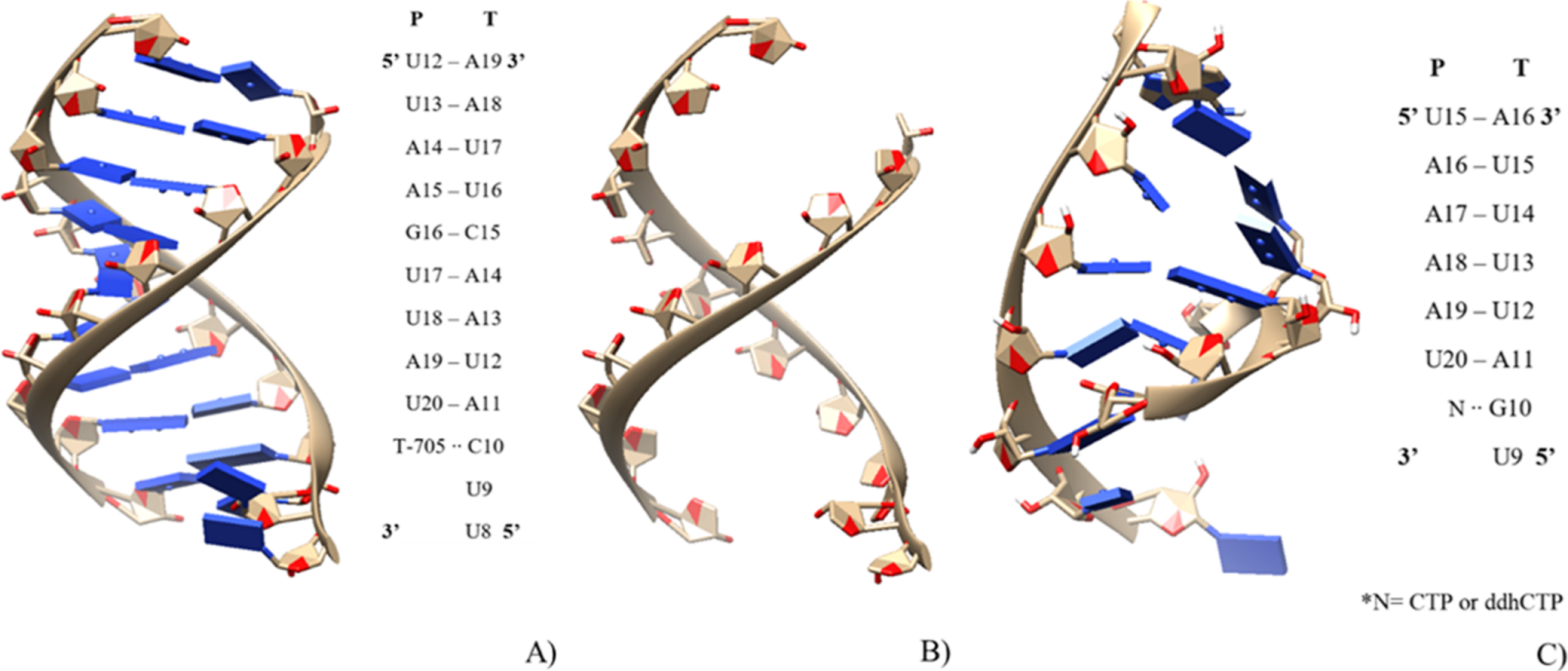
(A) Cryo-EM structure of favipiravir−RTP in the catalytic site of the SARS-CoV-2 RdRp, in complex with the template:primer RNA and 2D representation of the template (T) and primer (P) including T-705 (favipiravir). (B) Structure of favipiravir−RTP at the catalytic site of the RdRp, in which only the phosphate ribose scaffold is retained from the pdb 7AAP. (C) Computational model used for RdRp in complex with the template:primer RNA and 2D representation of the template (T) and primer (P) incorporating the ddhCTP inhibitor.

To obtain the desired model, the phosphodiester bond strain has been retained from the starting cryoEM structure, while all the already present nucleobases have been scrupulously replaced in silico, as shown in Figure 2, in agreement with the sequence for pyrimidine-like RdRp inhibitors. The tleap tool in Amber16^39^ has been used to obtain the final model (Figure 2C).

### 2.2 MD Simulations Setting

The parameters of CTP were obtained by adopting AMBER 94 and AMBER 99 force field^40^ data of mono-phosphate cytidine, and parameters for the two-terminal phosphate group of adenosine triphosphate are available in the AMBER parameter database. In order to obtain ddhCTP parameters, gas-phase geometry optimization has been carried out using B3LYP/6-311G*. Atomic charges were derived by fitting the electrostatic potential according to the Merz–Singh–Kollman scheme,^41^ using the RESP procedure. Antechamber and parmchk modules of Amber16 have been employed to generate preparatory files to perform molecular mechanics (MM) relaxation of the complexes. 20 Na^+^ counter ions were added to neutralize the system for both RdRp:RNA:ddhCTP and RdRp:RNA:CTP cases.

The RdRp RNA protonation state was prepared at physiological pH by using the H^++^ server,^42^ with a salt concentration of 0.15 M.^37^ The Amber force fields FF14SB^43^ and RNA.OL3^44^ were used for the protein and RNA, respectively. The ZAFF force field,^45^ specifically parameterized for zinc-containing systems in its common coordination in proteins, was selected for two Zn^2+^ ions, each of them coordinated to three Cys units and one His unit, as specified above. These ions only play a structural function. The Mg ions were treated with the Li et al. force field.^46^ It is well-known that classical force field parameters for Mg^2+^ ions are challenged in MD simulations with RNA filaments. Our choice follows the guidelines on site-specific structural features for Mg^2+^ ions in RdRp:RNA, as suggested by Casalino et al.^47^ and in agreement with other computational polymerase studies.^48,49^ The system was solvated in a orthorhombic box (12 Å from the protein) of TIP3P water molecules.^50^

The solvated structures were first minimized by applying harmonic restraints on all atoms of the enzyme (50 kcal/mol Å^2^), using 5000 steps of the steepest descent algorithm, followed by 5000 steps of the conjugate gradient algorithm. In the second minimization step, we released the restraint on hydrogen atoms, with the third and fourth minimizations being conducted with and without restraints to the protein backbone atoms, respectively. We carried out a progressive heating phase from 0 to 310 K for 200 ps using the Langevin thermostat in the *NVT* ensemble, with a time step of 0.002 ps. The production phase of 300 ns for RdRp:RNA and that of 300 ns for both RdRp:RNA:ddhCTP and RdRp:RNA:CTP complexes were performed under an integration step of 2 fs using the coupling SHAKE algorithm with the *NPT* ensemble at 1 bar pressure using the Berendsen barostat.^51^ The particle mesh Ewald summation method^52^ was employed for the electrostatic potential, and the long-range electrostatic interactions were calculated with the 12 Å cutoff distance. Trajectories for RdRp:RNA and its complexes with ddhCTP and CTP were saved every 0.2 ps and analyzed through the *ptraj* module.^53^ Overall, 0.9 μs of simulations was conducted (300 ns for each complex).

MD trajectories thus obtained can then be used to assess the magnitude of structural changes in terms of the root mean square deviation (RMSD), the propensity for a given residue or region to move, the root mean square fluctuation (RMSF), and the evolution of hydrogen bonding networks. The secondary structures were assigned using the DSSP algorithm.^54^

The RMSD-based clustering of the whole trajectories was performed according to the relaxed complex scheme protocol, as implemented in Amber16,^39^ to provide a sampled and energetically accessible conformational ensemble. After removing overall rotations and translations by RMS fitting the Cα atoms’ positions of the trajectory, the average linkage clustering algorithm was applied as implemented in *cpptraj* to identify 10 representative conformation clusters of the protein. The four most populated structures were used as a starting point for the docking procedure. The described procedure has been successfully adopted in a number of investigations on different enzymes.^55−57^

Principal component analysis (PCA) was performed using the *cpptraj* module of Ambertools 16 to extract the large-scale collective motions occurring in MD simulations of RdRp:RNA, RdRp:RNA:CTP, and RdRp:RNA:ddhCTP.^39^

Binding free energies between the binary complex RdRp:RNA and ddhCTP or CTP were calculated by solving the linearized Poisson–Boltzmann equation using the MM Poisson–Boltzmann surface area (MM-PBSA) method, as implemented in the Amber16 code,^39^ selecting the per-residue decomposition scheme. The value of the *igb* flag equal to 5 associated to a salt concentration of 0.1 M was used. For the calculations, 200 frames from each MD trajectory in the last 100 ns were analyzed.

## 3 Results and Discussion

### 3.1 MD Simulations of the RdRp:RNA Complex

As the initial part of the investigation, 300 ns of the MD simulation on the RdRp:RNA complex was performed. The RMSD plot calculated for all the backbone atoms of the amino acid residues shows that the system reached the equilibrium after a few nanoseconds of the simulation, as confirmed by the calculated average value of 1.49 ± 0.21 Å (see Figure S1). Such an observed behavior can be further evinced by the visual inspection of the protein structures obtained from the hierarchical clustering procedures (see Figure S2).

In addition, the RMSF of each individual residue of the non-structural proteins and RNA was calculated to characterize the local structural fluctuations in each subunit (Figure S3). High peaks were observed at Val405, Lys430, Thr644, Val737, and Thr850, which corresponded to loop regions of the protein, and at Leu895 and Glu903 in the proximity of carboxyl ends. Altogether, the preliminary analysis of the set of results on the structural stability of the RdRp:RNA complex allowed us to consider the in silico designed model to be reasonably stable.

**Figure 3:**
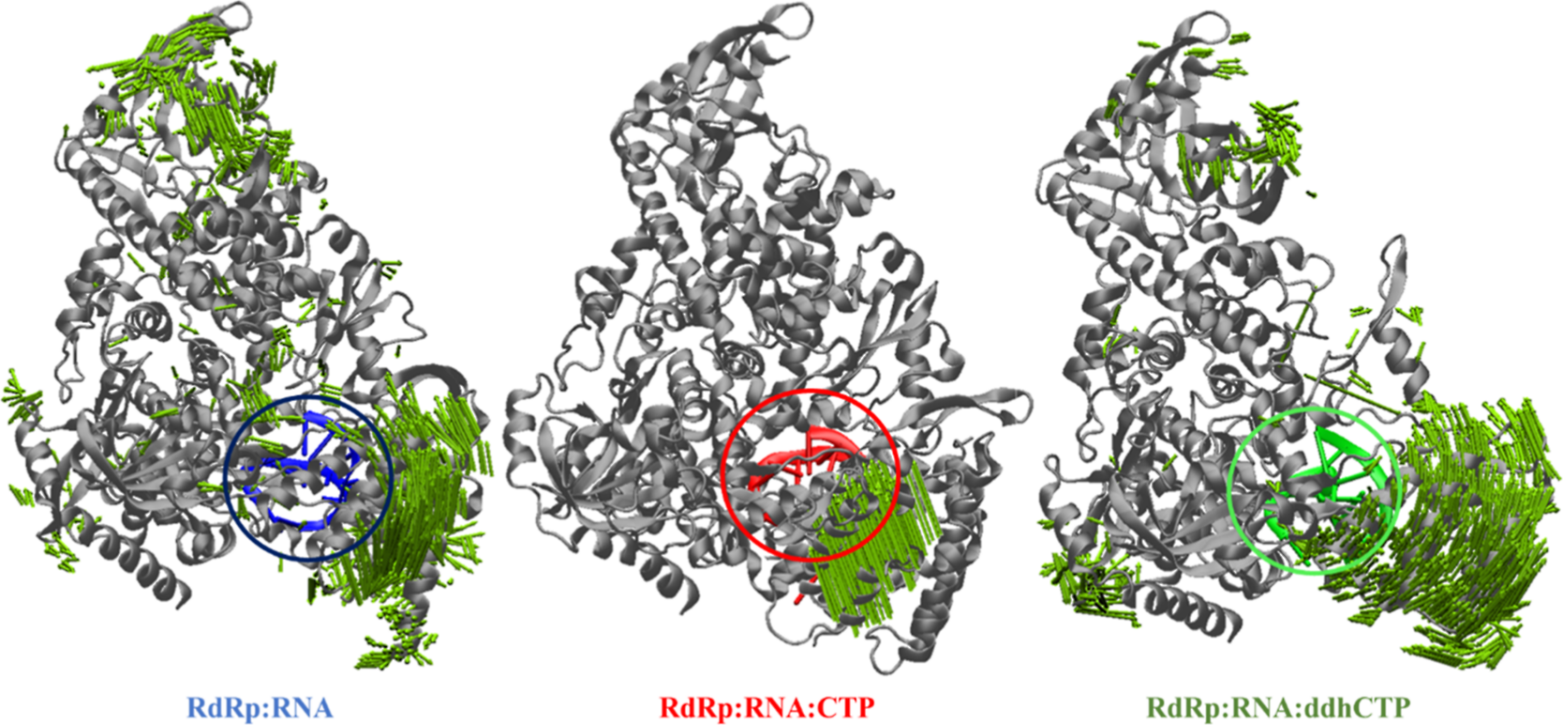
PCA of the RdRp:RNA binary, RdRp:RNA:CTP, and RdRp:RNA:ddhCTP ternary complexes. In every system, the RNA binding site is circled. The most mobile portions are highlighted by the green arrows.

PCA applied to MD trajectories to disclose the displacement of nsp7, nsp8, and nsp12 domains along the simulation has been carried out. The domains nsp7 and nsp8 are those that are offset more during the simulation, with respect to the nsp12 one (see Figure 3). However, the present investigation does not consider such an event, which is not therefore the object of the study. Indeed, although nsp7 and nsp8 were retained in the simulations, we focused our attention on the catalytic subunit nsp12. The structural stability of the RNA chains is not affected by the above-mentioned movement, as confirmed by the well-embedded conformation of RNA in the nsp12 pocket (Figure 3). The visual inspection of PCA calculated for the RNA, furthermore, highlights that a slight shift takes place during the simulation for the nucleic acid, mainly in the 3′ and 5′ positions of the T nucleotides (see Figure 3).

The structural homogeneity of the nucleic acid is ensured by hydrogen bond interactions occurring with the positively charged residues of the domains, such as Arg569, Lys849, and Arg858 (Figure S4 and Table S1). Further high interaction occupancies occurred between P:U20, T:G10, T:U12, P:A17, and P:A18 and Asp760, Ser682, Arg569, Arg585, and Arg836, respectively, of the different subunits with an occupancy range of 77–100, as shown in Table S2 related to native contacts.

In the selected crystal structure, the active site Mg_A_ and Mg_B_ cations are far from putative ligands, such as aspartate or glutamate residues, hardly enabling the establishment of the metal first coordination sphere.^26^ However, the analysis of the trajectory revealed that Mg_A_ and Mg_B_ interacted mainly with Asp618, Asp760, and Asp 761, as is shown later in detail. In general, both cations resulted in an octahedral geometry, further consisting of solvent molecules, as interestingly evidenced by the analysis of radial distribution functions (RDFs) for the Mg_A_–O_W_ and Mg_B_–O_W_ pairs. In particular, the first and second hydration spheres for the two metal ions were identified, with higher peaks observed in the case of Mg_B_ (see Figure S5). The most frequent presence of waters in proximity of Mg_B_, with respect to Mg_A_, can be ascribable to the P:U20 nucleotide located in proximity of the latter, which occupies the room for solvent molecules. The presence of H_2_O molecules has been further confirmed by the visual inspection of structures collected from the hierarchical clustering analysis, supplying an average number of four and five molecules in proximity of Mg_A_ and Mg_B_, respectively (Table S3).

### 3.2 MD Simulations of RdRp:RNA:CTP and RdRp:RNA:ddhCTP Complexes

The average binding affinities (in kcal/mol) obtained for the best docking poses of ddhCTP and CTP into the SARS-CoV-2 RdRp are reported in Table S4. The most energetically favored conformers, −11.4 and −11.3 kcal/mol for of RdRp:RNA:CTP and RdRp:RNA:ddhCTP, respectively, confirm that the introduction of ddhCTP can be considered feasible and, thus, can be allowed.

**Figure 4:**
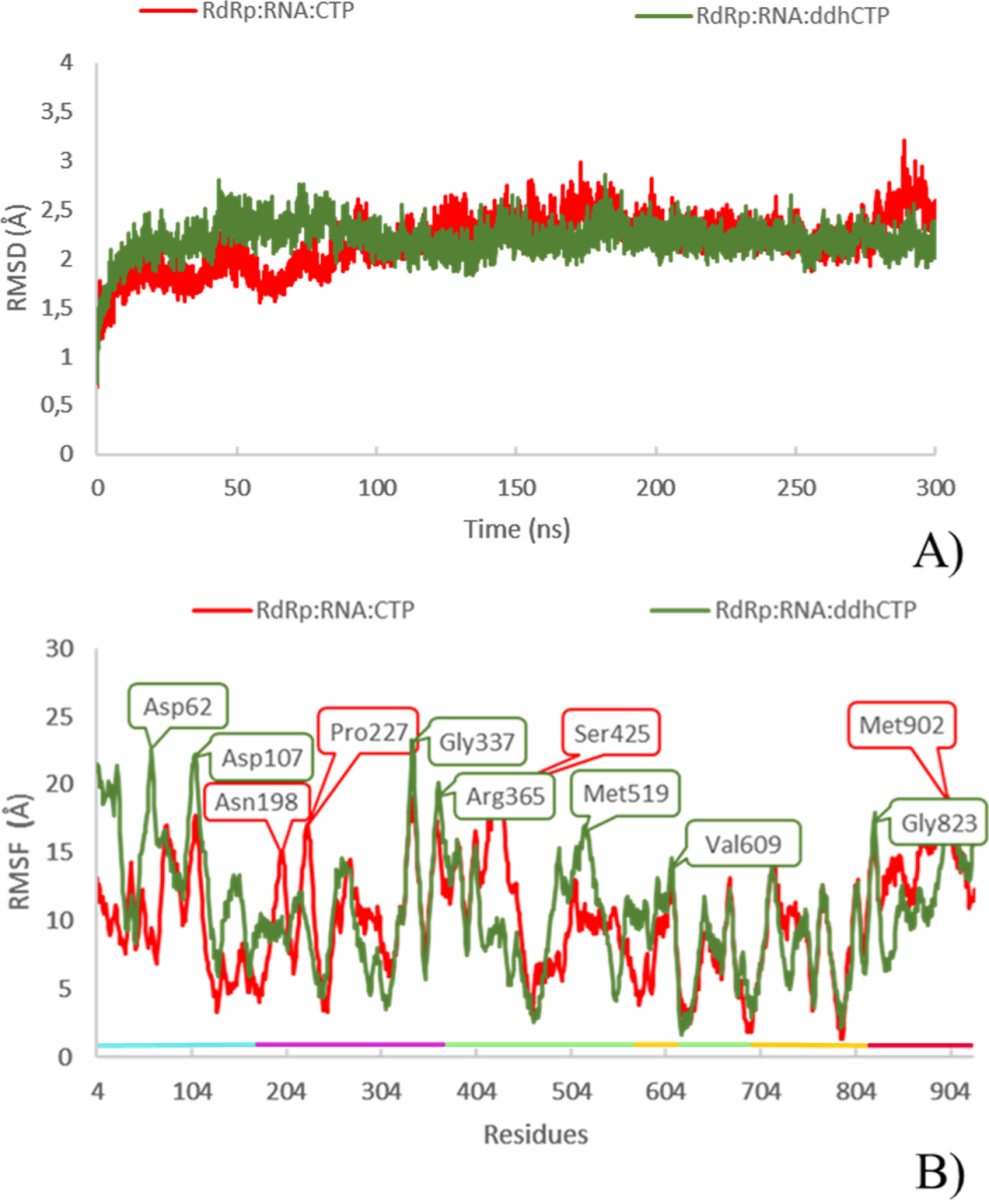
RMSD and RMSF plots of RdRP:RNA:CTP (red) and RdRP:RNA:ddhCTP (green) complexes.

After the docking of the ligands, 300 ns of MD simulations were further performed on both RdRp:RNA:CTP and RdRp:RNA:ddhCTP ternary complexes. The RMSD plot reveals that the system reached the equilibrium, with average values slightly higher than that of the respective RdRp:RNA system (2.18 ± 0.28 and 2.20 ± 0.17 Å for CTP and ddhCTP, respectively, versus 1.49 ± 0.21 Å for the binary, see Figure 4). Consistent with such a behavior, the calculated RMSF values of ternary complexes were also higher than the binary one (see Figure 4A).

In particular, in the case of the RdRp complexed to CTP, more intense fluctuations were detected in proximity of NiRAN (Asn198 and Pro227), finger (Ser425), and thumb (Met902) subdomains.

In the MD simulations of RdRp:RNA:ddhCTP, the major fluctuations were further observed in the interface subdomain, as evidenced by the peaks obtained for Gly337 and Arg365 residues exposed to the solvent, in addition to fluctuations for the NiRAN (Asp62 and Asp107) and finger (Met519 and Val 609) subdomains (see Figure 4B).

In analogy to the observation obtained for the RdRp:RNA complex, PCA revealed that for both ternary complexes, nsp12 and nsp8 are the regions of the system involved in more detectable movements during the MD simulations and that the RNA molecule conformation is not affected by any important structural rearrangement (see Figure 3).

In the binding site, CTP and ddhCTP ligands are stably located during the entire simulation, as can be observed by the analysis of their RMSD plots reported in Figure S6. The orientation of the substrates is mainly ensured by the presence of cation–phosphate interactions, occurring with different combinations between the phosphate arms of CTP and ddhCTP with Mg_A_ and Mg_B_. The distances of each Mg ions with P_α_, P_β_, and P_γ_ were analyzed to characterize the binding mode of the phosphate moieties of ligands during the simulation, and the relative distributions are displayed in Figure S7. In the case of CTP, the plot analysis revealed that oxygens of the P_α_ group bridge both cations, while those of P_γ_ coordinate mainly the Mg_B_ (see Figure S8 for further details). A different behavior of the P_α_ group can be observed in ddhCTP as far as the Mg_A_ is concerned. In fact, it is shifted at distances around 5 Å.

**Figure 5:**
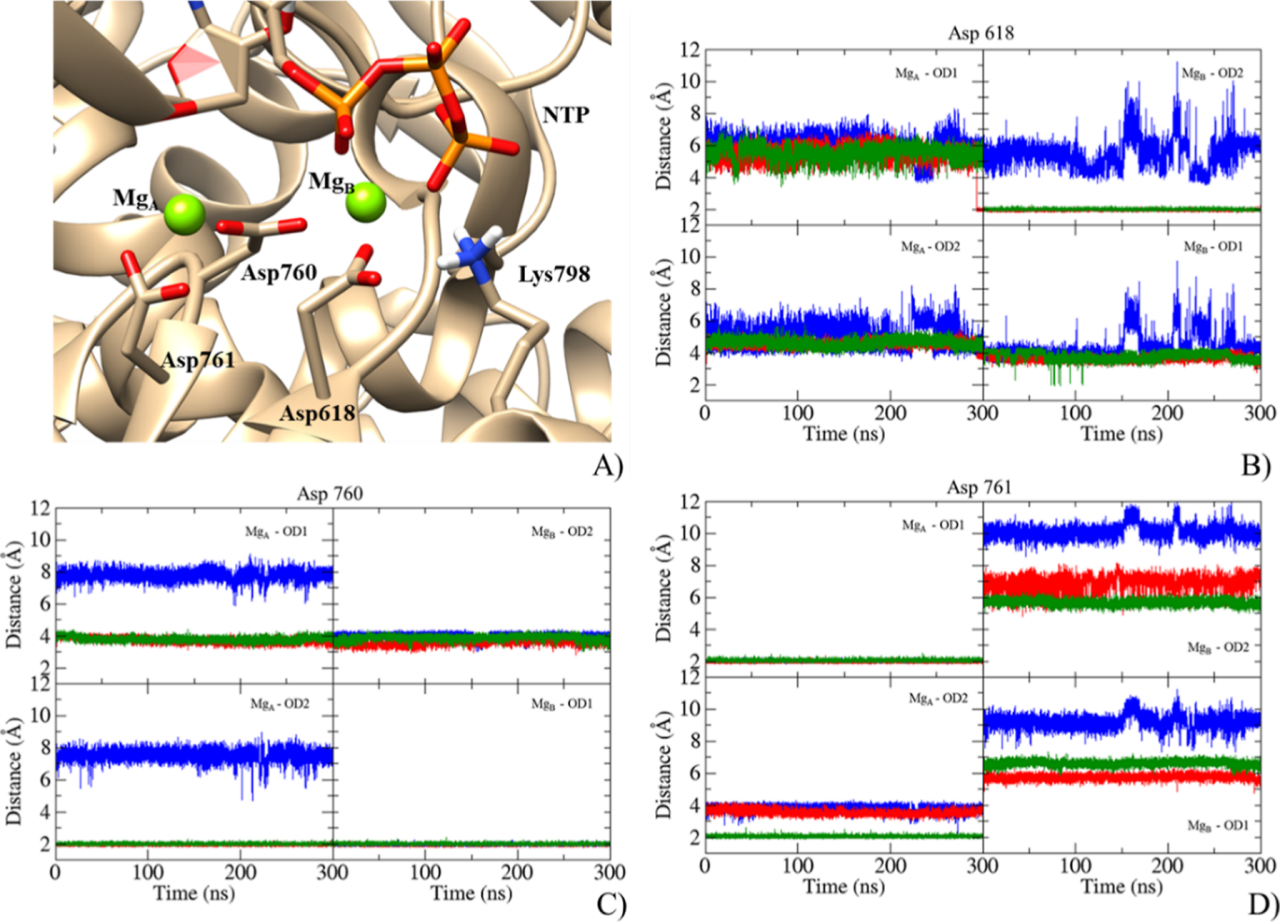
(A) Active site of the RdRp:RNA:NTP complex (NTP = CTP or ddhCTP). Distances calculated between MgA or MgB and (B) Asp618,(C) Asp760, and (D) Asp761 for the RdRp:RNA (blue), RdRp:RNA:CTP (red), and RdRp:RNA:ddhCTP (green) systems.

For Mg_B_, the distributions of P_α_ and P_γ_ results are very similar for both ligands, while P_β_ in ddhCTP is longer than that in CTP. In addition to the phosphate groups of CTP and ddhCTP, Mg_A_ and Mg_B_ complete their hexa-coordination (Figure S8) with the side chain of two consecutive Asp residues in motif C, Asp 760 in a μ(1,3) fashion, as confirmed by a measured distance of ca 2 Å for metal–O_Asp760_ distances (see Figure 5), and Asp761. Asp618, at the beginning of motif A, preferentially coordinates the Mg_B_ one.

The above-described orientation of P_α_, P_β_, and P_γ_ differs from that observed for remdesivir, in the active site of RdRp.^11^ It can be ascribable to a more advanced step of the recognition of remdesivir, considering that the OH of the primer is deprotonated and already coordinated to Mg_A_. However, the orientation described here of ddhCTP is encountered in other inhibitors, generally identified as “chain terminators” of SARS-CoV2 polymerase^16^ or UTP analogues of viral Bat Influenza A polymerase.^58^

The oxygen atoms of P_β_ interacted during the whole simulation with the positively charged Lys551 and Arg553 residues of motif F for both examined ternary complexes, thus contributing to the binding of CTP and ddhCTP. Our finding is in good agreement with the observations on other viral nucleic acid polymerases.^11,21,54,59,60^

**Figure 6:**
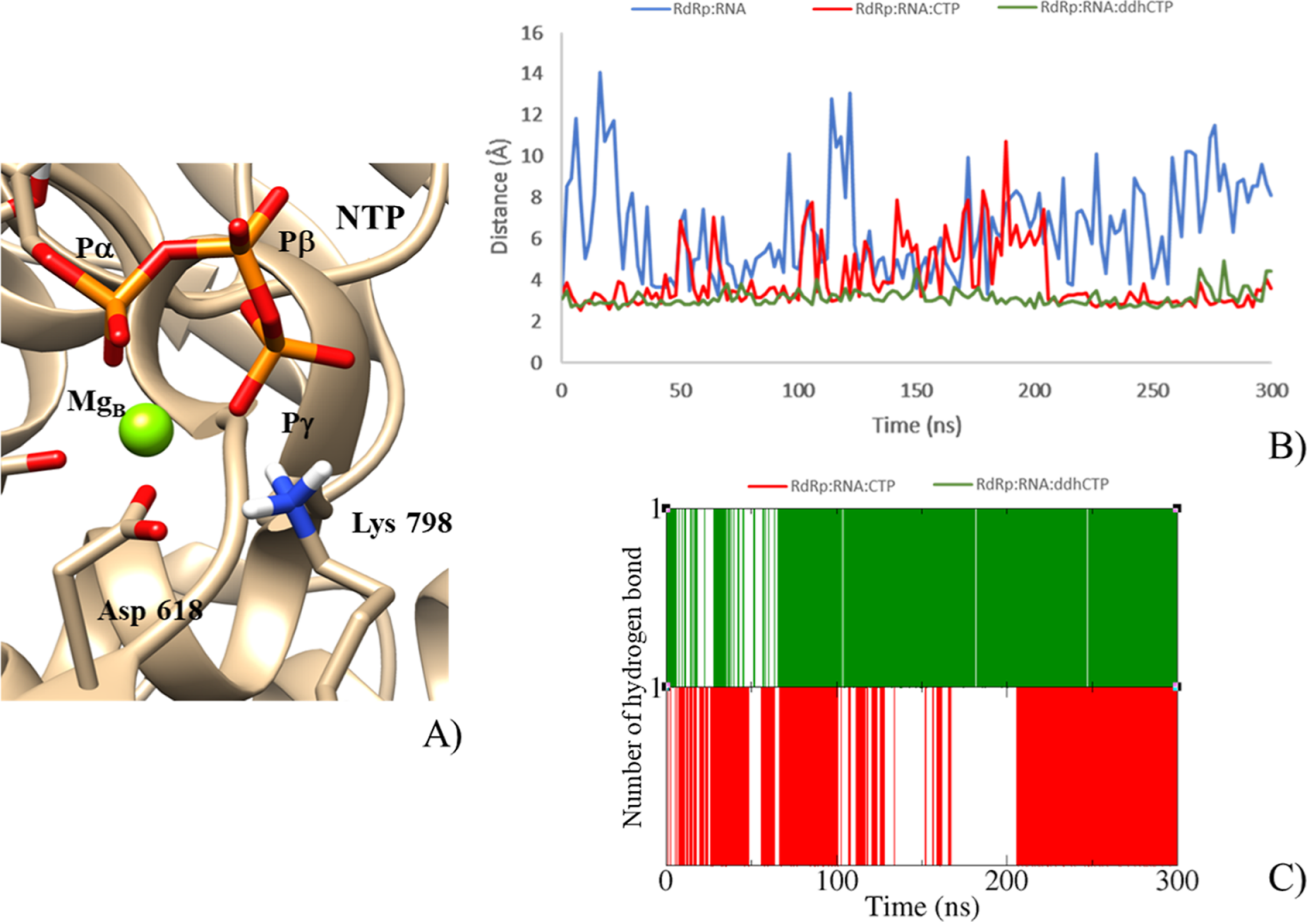
(A) Focus on the Lys798 orientation in the active site of the RdRP:RNA:NTP complex. (B) Asp618−Lys798 salt bridge distance and (C) OP_γ_−N_Lys798_ hydrogen bond calculated in the MD simulations of RdRP:RNA:NTP complexes (NTP = CTP or ddhCTP).

Very interestingly, the RdRp:RNA:CTP and RdRp:RNA:ddhCTP analyses of the trajectories of salt-bridges established by Asp618 revealed that it is frequently implicated in the interaction with Lys798, while the same interaction was not frequently observed in the binary complex (Figure 6).

This behavior highlights the role played by Lys798 as the “needed counterion” in orienting the P_γ_ toward Mg_B_. Lys798 is indeed located within the interactive hydrogen bond distance to the P_γ_ of incoming ligands, thereby suggesting that such a residue is involved in the binding of phosphorylated substrates and thus in the release of PPi after the chemical reaction has been completed (Figure 6).

This residue indeed represents the highly conserved lysine, critical in the control of the activity and fidelity of the transcription and in the action, acting as a general acid to protonate the pyrophosphate leaving group, as widely emphasized in the literature.^6,54^

Finally, a lower number of water molecules coordinating Mg_A_ and Mg_B_ was observed during the simulations due to the presence of phosphate groups, as can be noted from the count of solvent molecules coordinating the cations in the clustered geometries (see Table S3).

During the simulations, Mg_A_ and Mg_B_ lie at distances of 4.48 ± 0.12 and 4.96 ± 0.15 Å for CTP and ddhCTP, respectively (Figure S9). The observed value is consistent with that required by the reaction mechanism of two metal ions (<4 Å). It was suggested that during the reaction, a shrinking of this distance may occur, leading O3′ to become closer to Pα and consequently facilitating the nucleophilic attack. The little longer distance in ddhCTP than that in CTP could affect the O3′–P_α_ alignment for catalysis during the nucleophilic attack, in the reactive Michaelis–Menten complex.^51−65^

In order to analyze the interaction involving the cytosine nucleobase, which is the same for both ligands, attention was focused on three main geometrical parameters involving the pairing with guanosine nucleobase, such as C1′_CTP/ddhCTP_–C1′_G:T_, N1_CTP/ddhCTP_–N1_G:T_, and N3_CTP/ddhCTP_–C1′_G:T_. Outcomes on the analysis of these distances are linked to the fidelity of DNA polymerases^52,66^ and can be extended to RNA polymerase due to the many similarities between the two classes of enzymes.^67−69^

In the case of the CTP ligand, the analysis of the distribution distances revealed that the nucleobases lie in an unpaired-like conformation, which can be linked to the low fidelity behavior of the RNA polymerases accordingly to the mechanism. Indeed, the calculated average values of 11.73 ± 0.47, 6.72 ± 0.59, and 5.98 ± 0.10 Å for C1′_CTP_–C1′_T:G_, N1_CTP_–N1_T:G_, and N3_CTP_–N1_T:G_ fit results described in a previous work.^48^ More interestingly, the inclusion of ddhCTP led to an increase of ca 2 Å for all the considered distances (see Figure S10), thus suggesting that in the presence of the endogenous inhibitor, the fidelity of the enzyme is further affected and that errors in the course of the duplication can take place already in this step, thus concurring to the stalling of the enzyme.

Due to the modification present at the 3′ position of the ribose ring of ddhCTP, the analysis of the RDF and visual inspection of clustered structures were conducted on the interactions involving the ribose group, focusing on the hydroxyl groups at C2′ and C3′ positions of CTP in comparison with that of ddhCTP. In the case of the CTP molecule, the O3′ atom established hydrogen bonds with the phosphate group of U20 and water molecules, while O2′ was mainly involved in interactions with solvent molecules. In the absence of O3′–H group in the ddhCTP-containing system, the interaction with U20 phosphate was observed to occur with the O2′ atoms (Figure S11).

From the contact analysis, effected for both CTP and ddhCTP, it can be evinced that residues 618−621 (motif A, 760 (motif C) and 551 (motif F) are implicated in interactions with the ligands (see Tables S5−S7), suggesting a similar interaction network in the binding site. However, the analysis did not provide any relevant information about the interactions involving the ribose hydroxyl groups and the amino acid residues. The calculation of Δ*G*_bind_, carried out according to the MM-PBSA scheme revealed some differences for the ligands. In detail, the binding free energy of ddhCTP was determined to be 15 kcal/mol higher than that of CTP (Table S8).

Energy decomposition analysis of every residue involved in non-covalent interaction with CTP and ddhCTP showed that, for the former, a stronger stabilization of the tertiary complex is observed due to the electrostatic interaction of Arg553, Arg555, Lys621, and Asp761 side chains (see the Supporting Information for details, Figure S12) with the active site environment.

In addition, the higher Δ*G*_bind_ (lower affinity) can be linked to the absence of the hydroxyl group at the C3′ position, which causes a loss of three hydrogen bond interactions from ddhCTP species. In the course of the dynamics, both CTP and ddhCTP, finally, assume a reacting like conformation. This can be evinced, in particular, by analyzing the Mg_A_–O3′P:U20 and the O3′P:U20–P_αNTP_ distances, which are the reaction coordinates involved in the reaction catalyzed by the enzyme.

**Figure 7:**
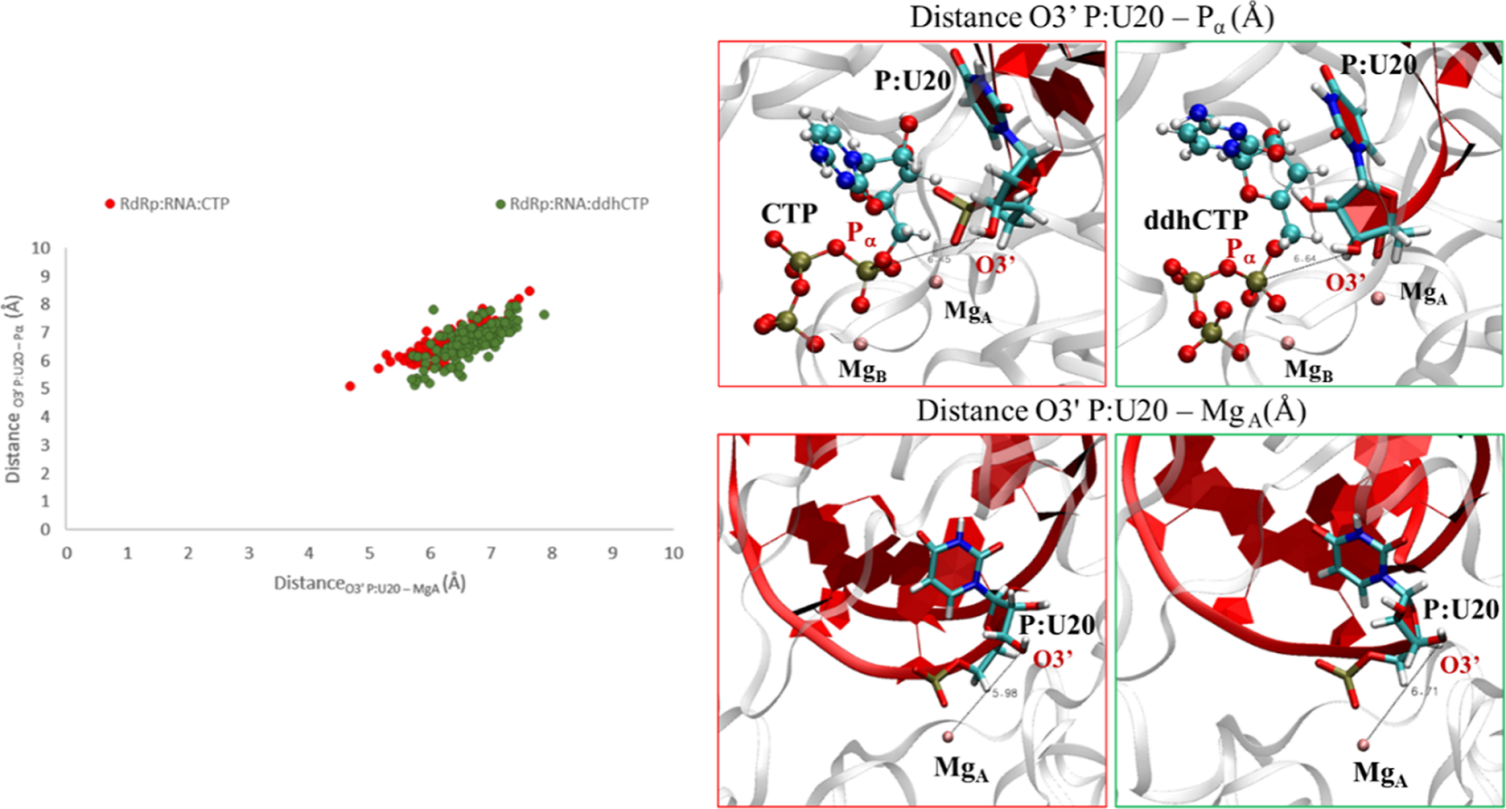
Distribution of O3′P:U20−P_α_ vs O3′P:U20−MgA distances calculated for RdRp:RNA:CTP and RdRp:RNA:ddhCTP complexes.

A very similar distribution of values was observed, see Figure 7, which can be further linked to the experimental evidence concerning the incorporation and reactivity in the presence of the ddhCTP ligand.^16^

## 4 Conclusions

Nucleotide analogues represent a powerful tool for fighting the viral infections and their consequences. In the present work, the interaction of the RdRp protein, involved in the spreading of the SARS-CoV-2 disease, and the endogenously synthesized ddhCTP, the product of the viperin antiviral protein, has been investigated. Such a molecule differs from CTP, its natural precursor, by the presence of a double bond and the absence of a 3′-OH group in the sugar ring. However, it is reputed that the missing hydroxyl group can favor the stalling of the enzyme and, consequently, the replication of viral RNA. The study has been conducted in the mean of all-atom MD simulations, on the RdRp:RNA system and on each RdRp:RNA:NTP complex, with NTP equal to CTP or ddhCTP, and has focused on the recognition step of the RdRp catalytic mechanism.

From the analysis of the binary complex, it is determined that the RNA is kept in the active site by interaction with a number of residues, such as Ser682 and Arg585, and that in the absence of other substrates, Mg_A_ and Mg_B_ present a hexa-coordination sphere, composed of Asp618, Asp760, Asp761, and water molecules.

The comparative analysis of the conformational behavior of ddhCTP and CTP, overall, indicated high similarity between the two molecules. In detail, phosphate groups P_α_ and P_γ_ of CTP and ddhCTP are directly involved in the coordination of Mg_A_ and Mg_B_, while P_β_ mainly interacted with the positively charged Lys551 and Arg553, facilitating the binding of the ligands in the active site. Importantly, Lys789 has been identified as a crucial residue bridging Asp618 and the P_γ_ group of both ddhCTP and CTP.

The hydroxyls groups of CTP at C2′ and C3′ positions established hydrogen bond interaction with solvent molecules and U20 of RNA, respectively, while in the case of ddhCTP, the interaction with nucleic acid is ensured by the 2′-OH group.

The interactions of the cytidine group of both nucleosides with guanine of the RNA chain were evaluated in terms of C1′_CTP_–C1′_T:G_, N1_CTP_–N1_T:G_, and N3_CTP_–N1_T:G_ distances. The analyzed results are in agreement with the low fidelity of RdRp, which was slightly more affected by the presence of the ddhCTP molecule. Finally, both CTP and ddhCTP showed pro-reactive conformation, with a good alignment of Mg_A_–O3′P:U20 and the O3′P:U20–P_αNTP_ distances observed for both the ligands.

The results described above shed light on the relevant aspects of the incorporation of CTP and ddhCTP species in the RdRp, laying the foundation for the rational design of the SARS-CoV2 vaccine and antiviral targets and providing further insights that were not accessible through experiments. Altogether, the available results from experiments and the rationalization from computational investigation can thus be very helpful for stimulating and driving future experimental investigations.

## Data and Software Availability

AutoDock version 4.2 was adopted to perform molecular docking calculations. All MD simulations were carried out using the AMBER16 package. The AmberTools package was further adopted in the preparation of the parameters and input for MD simulations. Density functional theory (DFT) calculations, to parametrize ddhCTP, were carried out using Gaussian 09 ver. D01. Trajectory analysis was performed using the AMBER cpptraj module. The preparation of the structures and the images was carried out using VMD software, version 1.9.3, and Chimera software. The full workflow is reported in the “Computational Methods” section of the manuscript.

## Supporting Information Available

Additional results of MD simulations of RdRp:RNA, RdRp:RNA:CTP, and RdRp:RNA:ddhCTP systems (PDF)
MD simulations of RdRp:RNA:CTP and RdRp:RNA:ddhCTP (ZIP)
