# Supplementary material for: On the Recognition of Natural Substrate CTP and Endogenous Inhibitor ddhCTP of SARS-CoV-2 RNA-Dependent RNA Polymerase: A Molecular Dynamics Study
Source: J Chem Inf Model. 2022 Oct 11;62(20):4916–27. doi: 10.1021/acs.jcim.2c01002 (PMC9597658; doi:10.1021/acs.jcim.2c01002)
Supplement: Supplementary file 1 — ci2c01002_si_001.pdf [file ci2c01002_si_001.pdf]

Supporting Information

On the recognition of natural substrate CTP and endogenous inhibitor ddhCTP of SARS-CoV-2 RNA-dependent RNA-polymerase: a molecular dynamics study.

Angela Parise[1,2], Giada Ciardullo[1], Mario Prejano[1], Aurélien de la Lande[2], Tiziana Marino[1]*

[1] Dipartimento di Chimica e Tecnologie Chimiche, Università della Calabria, Via Pietro Bucci, 87036 Arcavacata di Rende, CS, Italy

[2] Université Paris-Saclay, CNRS, Institut de Chimie Physique UMR8000, Orsay, France

*corresponding author: tiziana.marino65@unical.it

Figure S1. RMSD plot calculated for backbone atoms of binary RdRp:RNA complex..................S2

Figure S2. Superposition of binary RdRp:RNA complex, focusing on A) the whole system, A') RNA-free protein and A'') RNA chain. S3

Figure S3. RMSF plots of different chains, calculated for the RdRp:RNA complex. S3

Figure S4. Most representative structure from clustering analysis of the binary RNA:RdRp complex. Positive residues (Arg and Lys) in the binding pocket of RNA are evidenced. S4

Figure S5. RDF plot calculated for O_W-Mg_A and O_W-Mg_B pairs, for RdRp:RNA, RdRp:RNA:CTP and RdRp:RNA:ddhCTP systems. S5

Figure S6. RMSD plots of CTP and ddhCTP ligands. S6

Figure S7. Distance distributions of $P_{\alpha/\beta/\gamma}$-$Mg_{A/B}$ calculated for CTP and ddhCTP species, in the relative simulations. S6

Figure S8. Focus on active site composition of most representative clustered geometry of A) RdRp:RNA, B) RdRp:RNA:CTP and C) RdRp:RNA:ddhCTP complexes. S7

Figure S9. Distance distribution of Mg_A-Mg_B obtained from MDs of RdRp:RNA:ddhCTP and RdRp:RNA:CTP systems. S7

Figure S10. Distance distributions of $C1'_{CTP/ddhCTP}$-$C1'_{G:T}$, $N1_{CTP/ddhCTP}$ -$N1_{G:T}$ and $N3_{CTP/ddhCTP}$ -$C1'_{G:T}$ of both RdRp:RNA:ddhCTP and RdRp:RNA:CTP complexes. S8

Figure S11. RDF plots calculated for $C3'_{NTP}$-O_w pair during the MDs of RdRp:RNA:CTP and RdRp:RNA:ddhCTP systems (NTP=CTP or ddhCTP). S9

Figure S12. Plot of amino acid decomposition analysis, obtained from MMPBSA calculations. ..S9

Table S1. Results of evaluated contacts in the MD of RdRp:RNA system. S10

Table S2. Results of evaluated native contacts in the MD of RdRp:RNA system.........................S10

Table S3. Number of water molecules in proximity of Mg_A and Mg_B identified in the clustered geometries of RdRp:RNA, RdRp:RNA:CTP and RdRp:RNA:ddhCTP systems. S11
Table S4. Results of docking calculations. Values are in kcal/mol evaluated contacts in the MD of RdRp:RNA system. S11
Table S5. Results of calculated contacts of O2' hydroxyl group with nsp12 protein of RdRp:RNA:CTP system. S12
Table S6. Results of calculated contacts of O3' hydroxyl group with carboxylate groups of aspartate in the active site of RdRp:RNA:CTP system. S12
Table S7. Results of calculated contacts of O2' hydroxyl group with nsp12 protein of RdRp:RNA:ddhCTP system. S12
Table S8. Results of MMPBSA calculations. S13

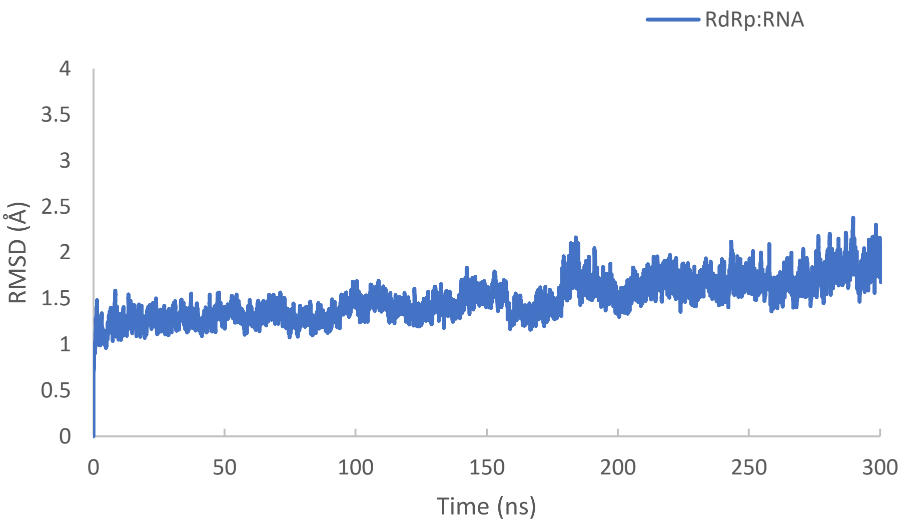

Figure S1. RMSD plot calculated for backbone atoms of binary RdRp:RNA complex.

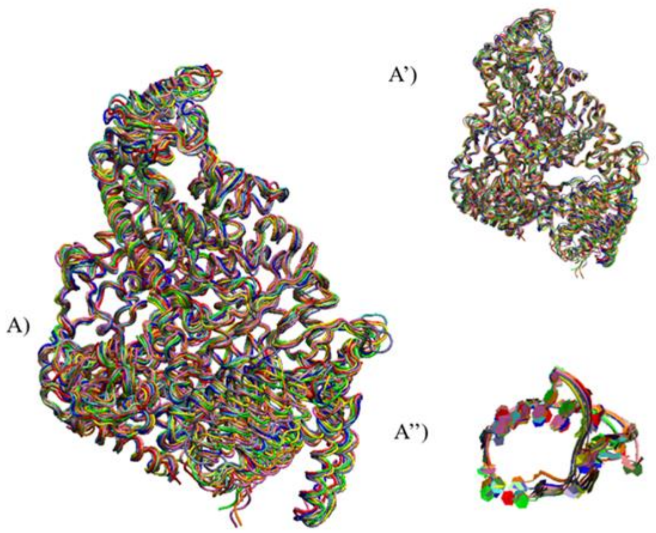

Figure S2. Superposition of binary RdRp:RNA complex, focusing on A) the whole system, A') RNA-free protein and A'') RNA chain.

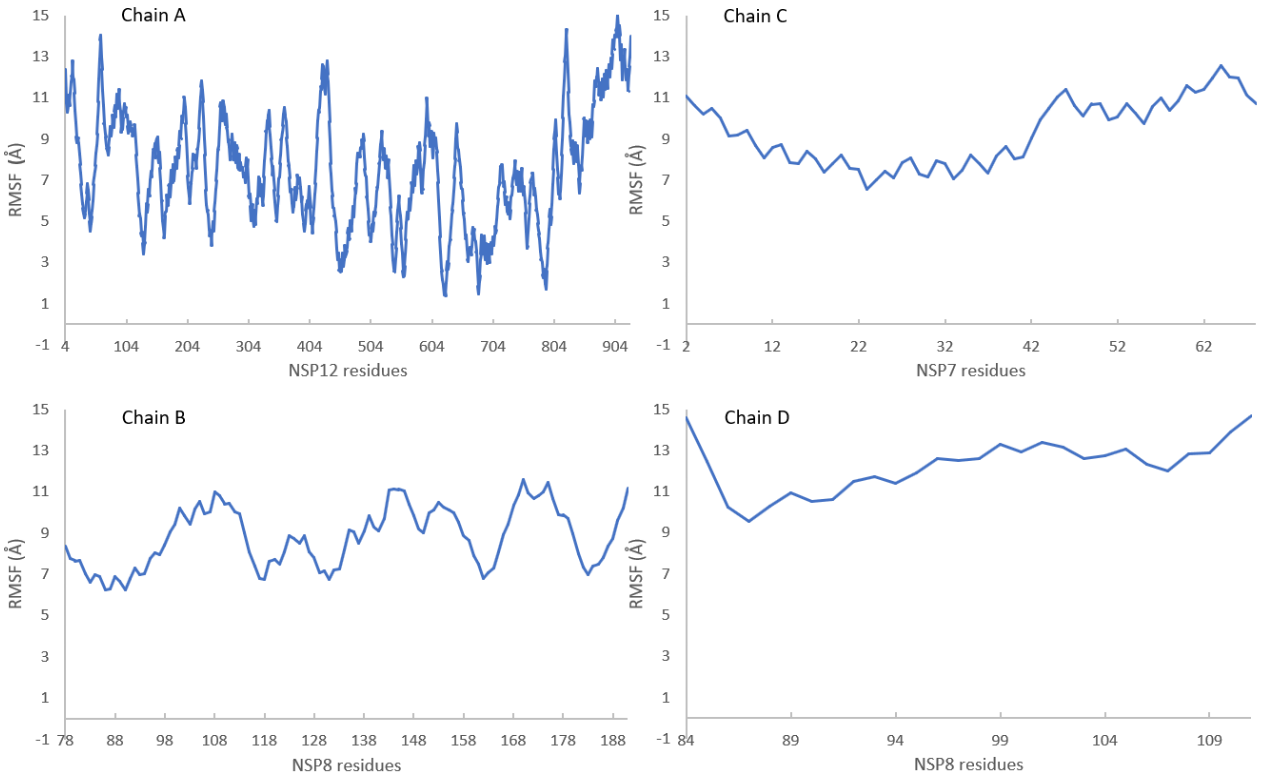

Figure S3. RMSF plots of different chains, calculated for the RdRp:RNA complex.

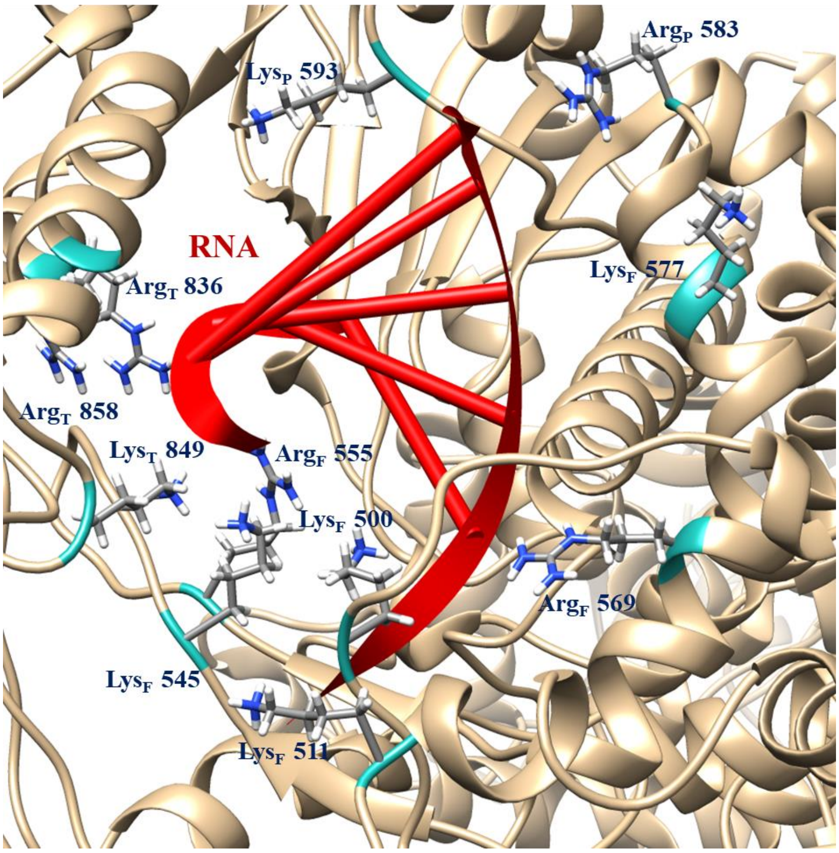

Figure S4. Most representative structure from clustering analysis of the binary RNA:RdRp complex. Positive residues (Arg and Lys) in the binding pocket of RNA are evidenced.

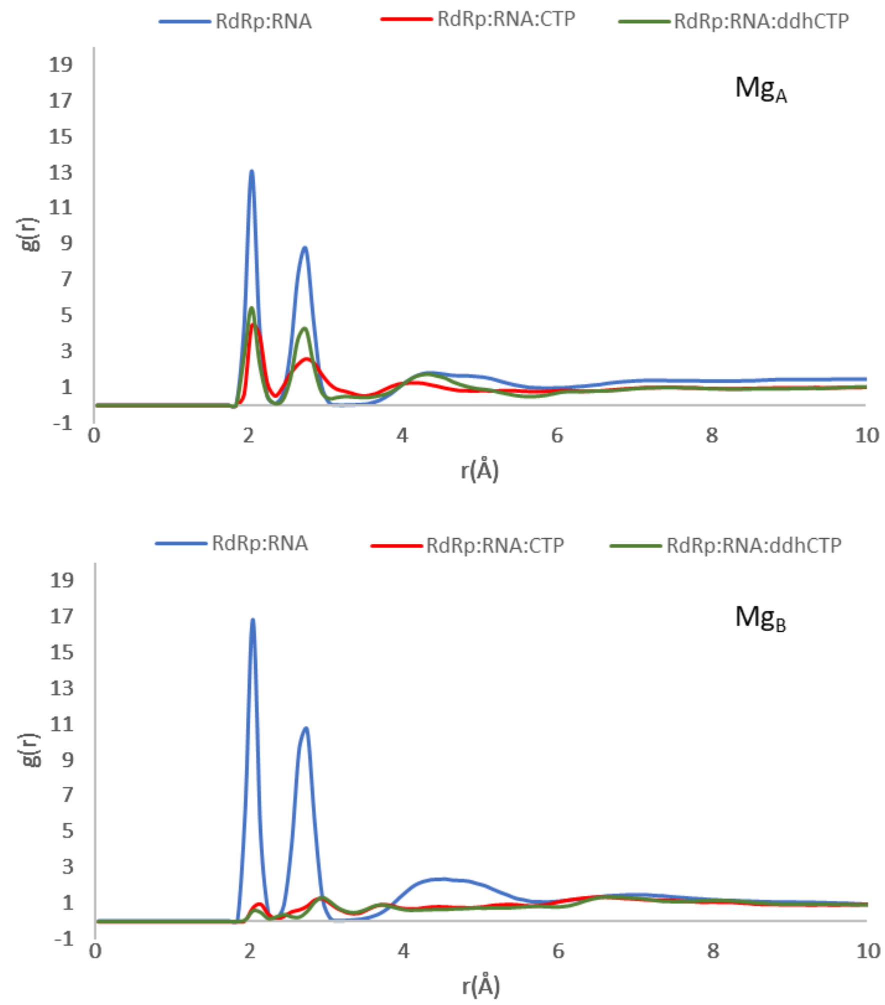

Figure S5. RDF plot calculated for O_W-Mg_A and O_W-Mg_B pairs, for RdRp:RNA, RdRp:RNA:CTP and RdRp:RNA:ddhCTP systems.

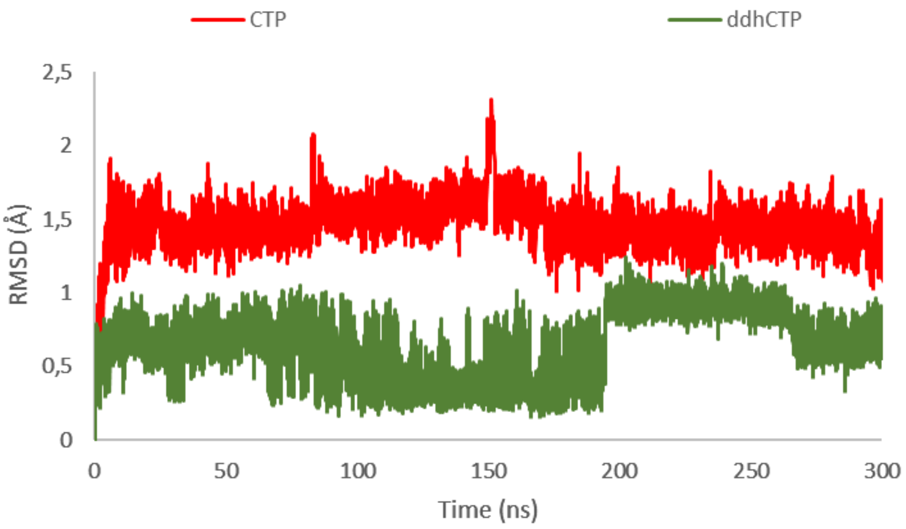

Figure S6. RMSD plots of CTP and ddhCTP ligands.

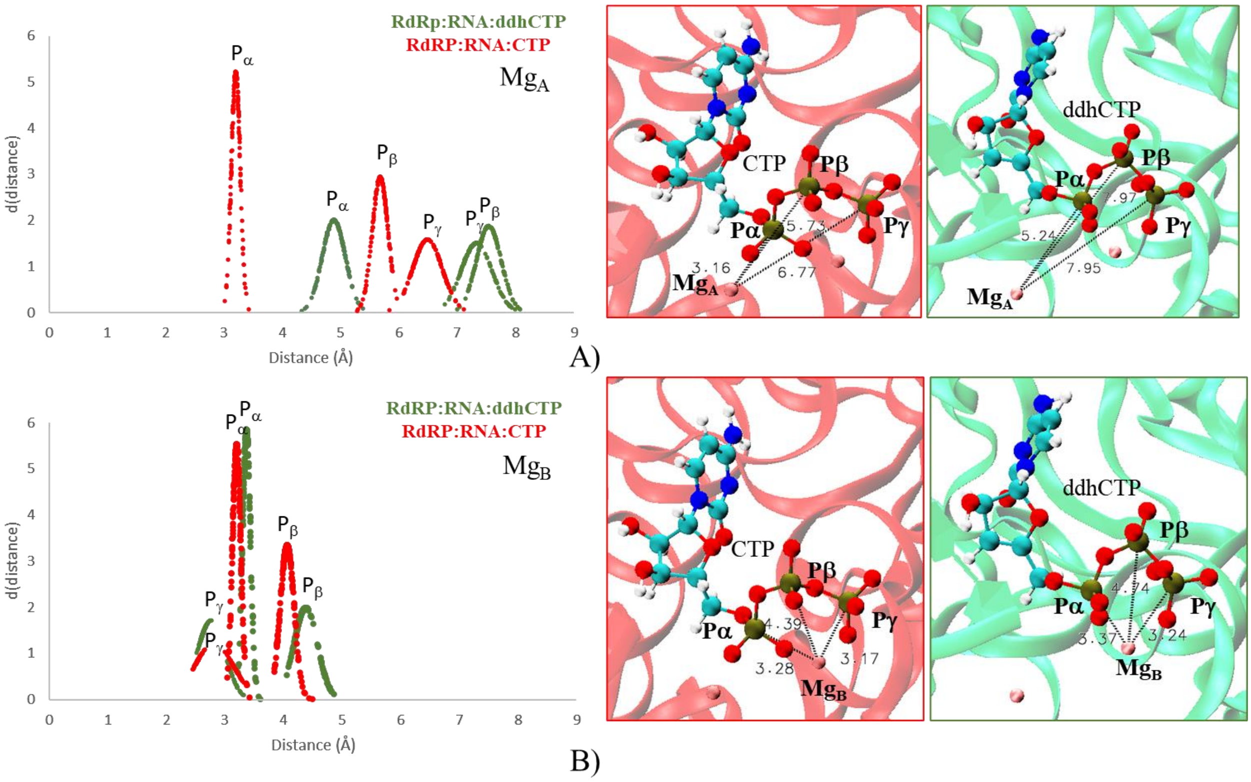

Figure S7. Distance distributions of $P_{\alpha/\beta/\gamma}$-$Mg_{A/B}$ calculated for CTP and ddhCTP species, in the relative simulations.

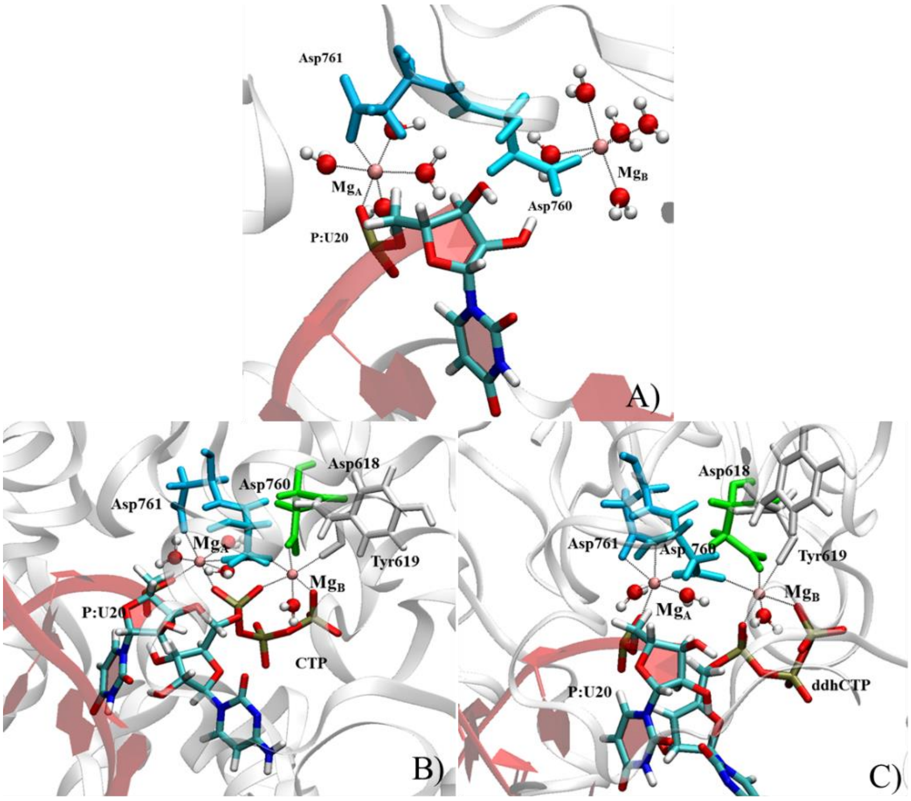

Figure S8. Focus on active site composition of most representative clustered geometry of A) RdRp:RNA, B) RdRp:RNA:CTP and C) RdRp:RNA:ddhCTP complexes.

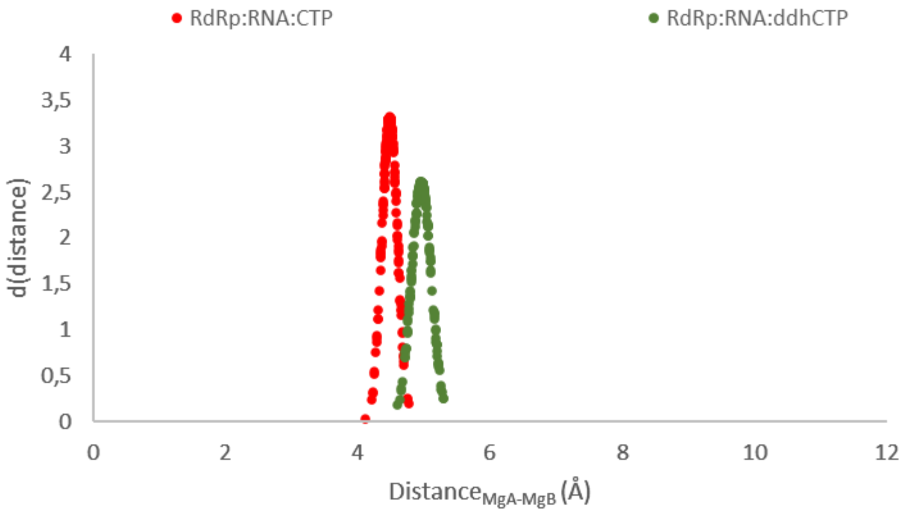

Figure S9. Distance distribution of Mg_A-Mg_B obtained from MDs of RdRp:RNA:ddhCTP and RdRp:RNA:CTP systems.

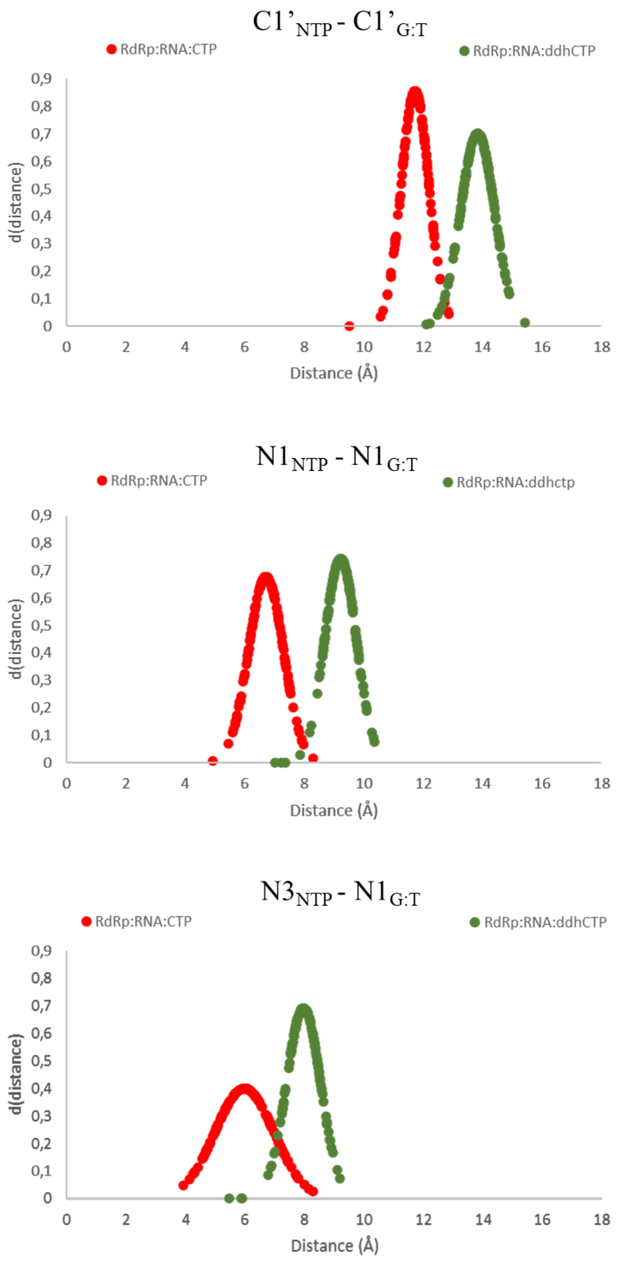

Figure S10. Distance distributions of C1'$_{CTP/ddhCTP}$-C1'$_{G:T}$, N1$_{CTP/ddhCTP}$ -N1$_{G:T}$ and N3$_{CTP/ddhCTP}$ - C1'$_{G:T}$ of both RdRp:RNA:ddhCTP and RdRp:RNA:CTP complexes.

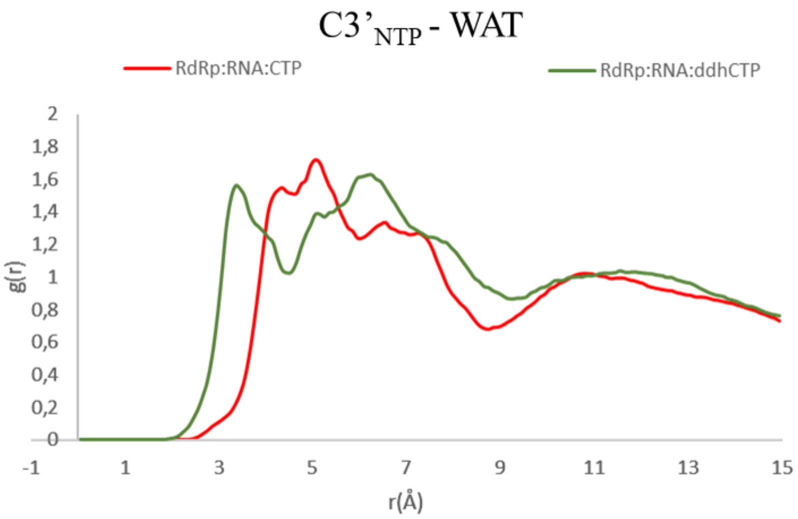

Figure S11. RDF plots calculated for C3'$_{NTP}$-O$_{W}$ pair during the MDs of RdRp:RNA:CTP and RdRp:RNA:ddhCTP systems (NTP=CTP or ddhCTP).

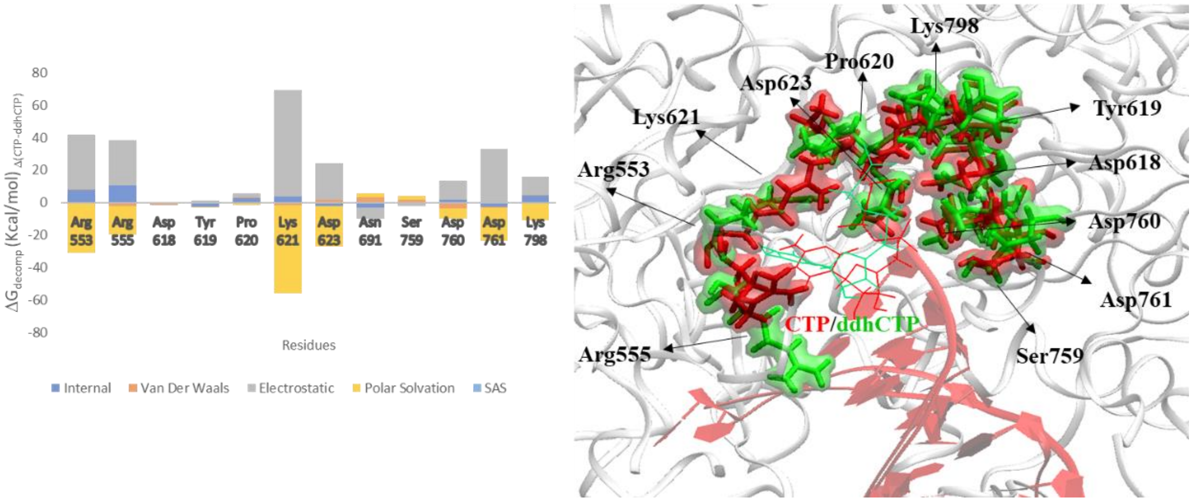

Figure S12. Plot of amino acid decomposition analysis, obtained from MMPBSA calculations.

Table S1. Results of evaluated contacts in the MD of RdRp:RNA system.

Acceptor	Donor	Frames	Frac	AvgDist	AvgAng
T:G10@OP1	SER 501@N	9520	63%	2,8781	161,8176
P:U20@O3'	ASP 760@N	8275	55%	2,8977	159,808
T:U14@O2'	SER 592@OG	7255	48%	2,799	160,2928
T:U13@P1	TYR 689@OH	6758	45%	2,8092	153,1448
T:U9@OP2	SER 501@OG	6296	42%	2,711	163,1832
T:U12@OP1	ARG 569@NE	6048	40%	2,8941	161,0654
T:U12@OP2	ARG 569@NH2	5198	35%	2,8603	158,7579
T:A11@OP1	ARG 569@NH2	4817	32%	2,8501	156,5644
T:U9@OP1	SER 501@OG	4787	32%	2,7101	164,842
P:A17@OP1	LYS 849@NZ	4247	28%	2,826	157,0055
T:U11@OP1	ARG 569@NH2	4141	28%	2,8705	155,9795
T:U9@OP1	ASN 507@ND2	4063	27%	2,8648	161,6711
P:A16@O2'	ARG 858@NH2	3769	25%	2,8819	152,3639

Acceptor	Donor	Frames	Frac	AvgDist	AvgAng
SER 679@O	T:G10@N2	13090	87%	2,8199	159,7903
ASP 760@OD2	P:U20@O2'	12280	82%	2,7486	159,1931
ASP 684@O	T:A11@O2'	10997	73%	2,7451	149,7018
ASP 865@OD1	P:A18@O2'	8190	55%	2,7264	165,871
ASP 865@OD2	P:A18@O2'	4228	28%	2,7289	165,8257

Table S2. Results of evaluated native contacts in the MD of RdRp:RNA system.

Contact	Nframes	Frac.	Avg	Stdev
P:U20@OP1 : ASP 761@OD1	15000	100%	2,88	0,0994
T:G10@N2 : SER 682@O	14987	100%	2,85	0,123
P:U20@O3' : ASP 760@N	14906	99%	3	0,14
T:G10@OP1 : SER 501@N	14899	99%	2,97	0,148
T:A11@O2' : ASP 684@O	14860	99%	2,79	0,162
T:G10@O2' : GLY 683@CA	14626	98%	3,11	0,144
P:A17@OP1: LYS 849@NZ	14618	98%	2,86	0,13
P:U20@O2' : ASP 760@OD2	13849	92%	2,79	0,155
T:U12@OP1: ARG 569@NE	13687	91%	3,05	0,177
P:A19@O3' : CYS 813@O	13369	89%	3,15	0,183
P:A19@O3' : CYS 813@C	13153	88%	3,25	0,134

T:U13@OP1 : TYR 689@OH	12416	83%	2,97	0,223
T:U14@C5' : GLY 590@O	12301	82%	3,22	0,148
T:U14@O2' : SER 592@OG	11975	80%	2,93	0,216
P:U20@O3' : SER 759@N	11574	77%	3,26	0,142

Table S3. Number of water molecules in proximity of Mg_A and Mg_B identified in the clustered geometries of RdRp:RNA, RdRp:RNA:CTP and RdRp:RNA:ddhCTP systems.

	**Number of water molecules within 3 Å of Mg_A**		
	**RdRp:RNA**	**RdRp:RNA:CTP**	**RdRp:RNA:ddhCTP**
**Cluster 0**	4	2	2
**Cluster 1**	4	2	2
**Cluster 2**	3	2	2
**Cluster 3**	4	2	2
**Cluster 4**	4	1	2
**Cluster 5**	4	2	2
**Cluster 6**	4	2	2
**Cluster 7**	4	2	2
**Cluster 8**	3	2	2
**Cluster 9**	4	2	2

	**Number of water molecules within 3 Å of Mg_B**		
	**RdRp:RNA**	**RdRp:RNA:CTP**	**RdRp:RNA:ddhCTP**
**Cluster 0**	5	1	1
**Cluster 1**	5	0	0
**Cluster 2**	5	0	0
**Cluster 3**	5	0	0
**Cluster 4**	5	1	0
**Cluster 5**	5	0	0
**Cluster 6**	5	1	0
**Cluster 7**	5	1	0
**Cluster 8**	5	0	0
**Cluster 9**	5	1	0

Table S4. Results of docking calculations. Values are in kcal/mol evaluated contacts in the MD of RdRp:RNA system.

**Docking pose**	**Docking score CTP**	**Docking score ddhCTP**

**1**	-11.4	-11.3
**2**	-9.1	-10.6
**3**	-8.5	-9.3
**4**	-3.0	-9.2

Table S5. Results of calculated contacts of O2' hydroxyl group with nsp12 protein of RdRp:RNA:CTP system.

Contact	Nframes	Frac	Avg	Stdev
CTP@O2':Lys545@CD	16	16%	5,56	0,37
CTP@O2':Val557@CG1	9	9%	5,46	0,511
CTP@O2':Ser682@CB	1	1%	4,12	0
CTP@O2':Asp623@OD2	1	1%	4,76	0
CTP@O2':Ser682@CA	1	1%	4,93	0
CTP@O2':Ser682@OG	1	1%	5,05	0
CTP@O2':Thr556@O	1	1%	5,15	0
CTP@O2':Val557@CB	1	1%	5,45	0
CTP@O2':Asp623@CG	1	1%	5,5	0
CTP@O2':Val557@CA	1	1%	5,67	0
CTP@O2':Ser682@N	1	1%	5,86	0
CTP@O2':Ser682@C	1	1%	5,89	.
CTP@O2':Ser682@O	1	1%	5,94	0
CTP@O2':Asp623@CB	1	1%	5,96	0

Table S6. Results of calculated contacts of O3' hydroxyl group with carboxylate groups of aspartate in the active site of RdRp:RNA:CTP system.

Contact	Nframes	Frac.	Avg	Stdev
CTP@O3':Asp623@OD2	1	1%	5,06	5,96E-08
CTP@O3':Asp623@CG	1	1%	5,63	5,96E-08
CTP@O3':Asp623@OD1	1	1%	5,78	8,43E-08

Table S7. Results of calculated contacts of O2' hydroxyl group with nsp12 protein of RdRp:RNA:ddhCTP system.

Contact	Nframes	Frac	Avg	Stdev

ddhCTP@O2:Arg555@NE	92	92%	4,41	0,875
ddhCTP@O2:Arg555@CD	92	92%	5,09	0,647
ddhCTP@O2:Arg555@CZ	87	87%	3,87	0,663
ddhCTP@O2:Arg555@NH1	87	87%	3,92	0,984
ddhCTP@O2:Arg555@NH2	83	83%	3,67	0,702
ddhCTP@O2:Arg555@CG	69	69%	5,31	0,65
ddhCTP@O2:Arg555@CB	18	18%	5,59	0,427
ddhCTP@O2:Ile548@O	9	9%	5,83	0,142

Table S8. Results of MMPBSA calculations. Solvation free energies are calculated using the Poisson–Boltzmann (PB) implicit solvent method with a nonpolar solvation term, based on the solvent accessible surface area (SASA) present in AMBER16. The vibrational frequencies of normal modes are calculated at various local minima of the potential energy surface.[1]

RdRp:RNA:ddhCTP

Energy Component	Average	Std. Dev.	Std. Err. of Mean
VDWAALS	-8.93	5.86	0.41
EEL	-512.02	53.56	3.78
EPB	434.11	52.03	3.67
ENPOLAR	-4.05	0.18	0.01
EDISPER	0.00	0.00	0.00
DELTA G gas	-520.96	52.50	3.70
DELTA G solv	430.06	51.94	3.66
DELTA TOTAL	-90.90	13.27	0.94

RdRp:RNA:CTP

Energy Component	Average	Std. Dev.	Std. Err. of Mean
VDWAALS	-1.18	6.60	0.47
EEL	-670.23	64.83	4.57
EPB	569.79	60.94	4.30
ENPOLAR	-4.23	0.11	0.01
EDISPER	0.00	0.00	0.00
DELTA G gas	-671.41	64.97	4.58
DELTA G solv	565.56	60.93	4.30
DELTA TOTAL	-105.85	14.10	0.99

1. Case, D. A.; Ben-Shalom, I. Y.; Brozell, S. R.; Cerutti, D. S.; Cheatham, T. E., III; Cruzeiro, V. W. D.; Darden, T. A.; Duke, R. E.; Ghoreishi, D.; Gilson, M. K.; Gohlke, H.; Goetz, A. W.; Greene, D.; Harris, R.; Homeyer, N.; Izadi, S.; Kovalenko, A.; Kurtzman, T.; Lee, T. S.; LeGrand, S.; Li, P.; Lin, C.; Liu, J.; Luchko, T.; Luo, R.; Mermelstein, D. J.; Merz, K. M.; Miao, Y.; Monard, G.; Nguyen, C.; Nguyen, H.; Omelyan, I.; Onufriev, A.; Pan, F.; Qi, R.; Roe, D. R.; Roitberg, A.; Sagui, C.; Schott-Verdugo, S.; Shen, J.; Simmerling, C. L.; Smith, J.; Salomon-Ferrer, R.; Swails, J.; Walker, R. C.; Wang, J.; Wei, H.; Wolf, R. M.; Wu, X.; Xiao, L.; York, D. M.; Kollman, P. A. AMBER 2017; University of California, San Francisco, **2017**.
